# Biosorption of Pb(II), Cu(II), and Zn(II) from aqueous solutions using unmodified coconut husk: characterization, adsorption equilibrium, and kinetic studies

**DOI:** 10.3389/fchem.2026.1889310

**Published:** 2026-07-27

**Authors:** Leonard Ejimofor Okonkwo, Hannah Ndidiamaka Okorie, Linda Nkechinyere Umegbo, Gerald Walter Ugodi

**Affiliations:** Faculty of Pharmaceutical Sciences, Enugu State University of Science and Technology, Agbani, Enugu, Nigeria

**Keywords:** adsorption isotherm, adsorption kinetics, biosorption, coconut husk, copper, environmental remediation, heavy metals, lead

## Abstract

**Introduction:**

Heavy-metal contamination of water and wastewater is a persistent environmental challenge because metallic pollutants are non-biodegradable, bioaccumulate through food chains, and pose serious risks to ecosystem and human health even at low concentrations. Conventional treatment technologies are often limited by high cost, secondary waste generation, and operational complexity, creating demand for low-cost, locally available alternatives. In this study, unmodified coconut husk was evaluated as a low-cost biosorbent for the removal of Pb(II), Cu(II), and Zn(II) from single-metal aqueous solutions.

**Methods:**

The biosorbent was characterized by Fourier-transform infrared spectroscopy (FTIR) and scanning electron microscopy (SEM). Batch adsorption experiments were conducted to investigate the effects of initial metal concentration (300–1,500 mg/L), adsorbent dose (0.2–1.0 g), solution pH (2.5–6.5), and contact time (5–120 min) on metal removal. Percentage removal increased with rising pH, adsorbent dose, and contact time, while it declined with increasing initial metal concentration.

**Results:**

Pb(II) consistently exhibited the highest adsorption affinity. Equilibrium data were better described by the Langmuir isotherm (R^2^ = 0.9808–0.9987) than by the Freundlich model, indicating predominantly monolayer adsorption; all Langmuir separation factor (R_L) values fell within the favourable range (0 < R_L < 1). Adsorption kinetics conformed to the pseudo-second-order model (R^2^ = 0.9694–0.9989) for all three metals, consistent with a chemisorption rate-limiting mechanism.

**Discussion:**

These results demonstrate that unmodified coconut husk is a promising, environmentally friendly, and economically accessible biosorbent for heavymetal removal and may have practical relevance for low-cost wastewater treatment in resource-limited settings.

## 1 Introduction

### 1.1 Heavy-metal contamination and the need for low-cost remediation

Heavy-metal pollution of aquatic environments represents one of the most significant challenges in environmental chemistry because metallic contaminants do not biodegrade and persist in water, sediment, and soil for extended periods (Bayuo et al., 2022; Jaishankar et al., 2014). Once introduced, toxic metals may undergo complex chemical transformations, partition between dissolved and solid phases, and enter food chains, ultimately reaching human populations through dietary exposure, inhalation, and dermal contact (Jaishankar et al., 2014; Okereafor et al., 2020).

Primary sources are overwhelmingly anthropogenic: electroplating, leather tanning, battery manufacturing, mining, smelting, and metal finishing discharge effluents containing Pb(II), Cu(II), and Zn(II) at concentrations that frequently exceed regulatory limits (Azimi et al., 2017; Duwiejuah et al., 2024). Lead is a non-essential element with a WHO maximum allowable limit of 0.01 mg/L in drinking water; chronic exposure causes neurological impairment, developmental delays, and renal dysfunction (Bayuo et al., 2022). Copper and zinc are essential micronutrients, yet become toxic above WHO guideline values of 2.0 mg/L and 3.0 mg/L, respectively, causing gastrointestinal, hepatic, and immunological damage (Bayuo et al., 2022; Jaishankar et al., 2014).

Conventional removal technologies—chemical precipitation, ion exchange, membrane filtration, and electrochemical treatment—are effective but constrained by high capital and operating costs, sludge generation, sensitivity to competing ions, and the need for skilled maintenance (Azimi et al., 2017; Shrestha et al., 2021). These limitations are especially acute in small and medium-scale operations in low- and middle-income countries, creating an urgent need for robust, easily deployable, low-cost alternatives (Bayuo et al., 2022; Gupta et al., 2021).

### 1.2 Adsorption and agricultural biosorbents

Adsorption is widely recognized as one of the most practical approaches for dissolved heavy-metal removal because of its operational simplicity, adaptability to batch and continuous systems, and compatibility with low-cost bio-based materials (Duwiejuah et al., 2024; Gupta et al., 2021). Activated carbon is the benchmark commercial adsorbent, but its high cost limits application in resource-constrained environments (Bayuo et al., 2022). This has driven extensive research into agricultural by-products—groundnut shells, banana peels, rice husk, corn cob, sugarcane bagasse, and coconut husk—which are abundant, renewable, and rich in oxygen-containing functional groups capable of binding metal cations via ion exchange, surface complexation, and electrostatic attraction (Duwiejuah et al., 2024; Gupta et al., 2021).

Coconut (*Cocos nucifera* L.) husk constitutes approximately 35% of the fruit by weight and is typically discarded as a low-value fibrous lignocellulosic residue (Malik et al., 2017). In Nigeria, it is often burned as fuel or left to decompose, representing both a waste management burden and a missed valorisation opportunity. From an adsorption perspective, coconut husk is attractive because its cellulose, hemicellulose, and lignin matrix provides a porous surface with hydroxyl, carboxyl, carbonyl, and ether groups capable of coordinating metal ions (Duwiejuah et al., 2024; Malik et al., 2017). Prior studies have demonstrated removal efficiencies of 60%–100% for various metals using raw or modified coconut husk (Duwiejuah et al., 2024; Hanafiah et al., 2020). The use of unmodified coconut husk avoids the cost, energy, and chemical inputs of activation, making it particularly practical for decentralized treatment in tropical regions where the material is locally abundant (Malik et al., 2017).

### 1.3 Emerging technologies for pollutant capture in water systems

The increasing occurrence of heavy metals, dyes, pharmaceuticals, personal care products, and other emerging contaminants in aquatic environments has stimulated the development of advanced water-treatment technologies beyond conventional adsorption systems. Among these, microfluidic extraction, membrane-based adsorption, electrocoagulation, reaction–diffusion-assisted processes, and hybrid treatment technologies have attracted considerable attention because of their high efficiency, reduced chemical consumption, and potential for sustainable environmental remediation (Shayesteh et al., 2021).

Microfluidic extraction systems represent a rapidly evolving approach for pollutant removal due to their enhanced mass-transfer characteristics and precise control of fluid flow at the microscale. Recent studies have demonstrated the effectiveness of microfluidic liquid–liquid extraction devices for the removal of organic pollutants and heavy metals from aqueous solutions (Raji et al., 2024). The small channel dimensions increase interfacial contact area and shorten diffusion distances, thereby improving extraction efficiency and reducing treatment time. Furthermore, computational fluid dynamics (CFD) modelling has been successfully employed to optimize channel-junction configurations and improve the sustainable extraction of heavy metals from contaminated waters (Toghyani et al., 2025).

Reaction–diffusion-based technologies have also emerged as promising tools for environmental remediation. The integration of dimensionless analysis and reaction–diffusion modelling in the continuous synthesis of alginate micro-gels has demonstrated improved dye-removal performance through enhanced control of particle formation and mass-transfer processes (Arefi et al., 2025). Such approaches provide valuable insight into pollutant transport mechanisms and contribute to the design of more efficient treatment systems.

Membrane-assisted adsorption represents another important advancement in water purification. Modified ion-exchange membranes and functionalized adsorbent surfaces have shown improved removal efficiencies for dyes and metal ions through the combination of selective transport and adsorption mechanisms. Enhanced methyl orange removal using modified anion-exchange membrane adsorbents demonstrates the potential of membrane-based systems for treating complex wastewater streams (Raji et al., 2023).

Electrocoagulation has gained increasing attention as an environmentally friendly treatment technology because it generates coagulant species *in situ*, thereby reducing chemical requirements (Sorayyaei et al., 2021). Hybrid electrocoagulation–adsorption systems have shown superior pollutant-removal performance compared with individual treatment processes by combining contaminant destabilization with adsorption onto solid surfaces (Babaei et al, 2025). Such integrated approaches have proven effective for the removal of dyes and other persistent pollutants from water (Mohammadi et al., 2023).

### 1.4 Process variables, modelling, and study objectives

A rigorous biosorbent evaluation requires systematic investigation of pH, initial metal concentration, adsorbent dose, and contact time, coupled with equilibrium and kinetic modelling (Bayuo et al., 2022; Reddad et al., 2002). Solution pH governs both surface protonation and metal speciation; initial concentration controls the mass-transfer driving force; dosage determines available binding sites; and contact time reveals rate-limiting steps (Gupta et al., 2021). The Langmuir and Freundlich isotherms are standard for interpreting equilibrium behaviour, while the pseudo-first-order and pseudo-second-order models describe adsorption kinetics. FTIR and SEM provide direct evidence of surface functional group participation and morphological changes associated with metal binding (El-Naggar et al., 2018; Rao, 2021).

Despite the advances achieved by these emerging technologies, adsorption using agricultural biosorbents remains attractive because of its simplicity, low operational cost, minimal energy requirements, and suitability for decentralized applications. Functionalized agri-waste biosorbents have demonstrated excellent adsorption capacities for Pb^2+^ and other toxic metals, highlighting the continuing relevance of biomass-derived materials in environmental remediation. Consequently, the investigation of abundant agricultural residues such as coconut husk remains important for developing sustainable and economically viable water-treatment solutions, particularly in resource-limited settings.

Despite growing literature on coconut-derived biosorbents, rigorous studies evaluating unmodified, locally sourced coconut husk for multi-metal assessment under controlled conditions—combining characterization, equilibrium modelling, and kinetic analysis—remain limited for the Nigerian context. This study therefore aimed to:

1. Characterize coconut husk before and after adsorption using FTIR and SEM;
2. Evaluate the effects of pH, initial metal concentration, adsorbent dose, and contact time on Pb(II), Cu(II), and Zn(II) removal in single-metal systems;
3. Apply Langmuir and Freundlich models to interpret equilibrium data;
4. Apply pseudo-first-order and pseudo-second-order models to describe adsorption kinetics; and
5. Assess the overall suitability of unmodified coconut husk as a low-cost biosorbent.

## 2 Materials and methods

### 2.1 Reagents and chemicals

All chemicals were analytical reagent (AR) grade, obtained from the Project Development Institute (PRODA), Enugu, Nigeria. Metal salts were zinc sulphate heptahydrate (ZnSO_4_ 7H_2_O), copper (II) sulphate pentahydrate (CuSO_4_ 5H_2_O), and lead (II) nitrate [Pb(NO_3_)_2_]. Potassium hydroxide (KOH, 0.1 M) and hydrochloric acid (HCl, 0.1 M) were used for pH adjustment. All solutions were prepared with distilled water.

### 2.2 Instrumentation

- pH measurement: Mettler Toledo SevenCompact pH/ion meter.
- Atomic absorption spectrophotometry (AAS): Buck Scientific Model 210 VGP AAS for residual metal determination.
- FTIR: Shimadzu FTIR-8400S spectrophotometer (KBr windows, 400–4,000 cm^-1^).
- SEM: Used to examine surface morphology before and after metal loading at 1,500×, 3,000×, and 5,000×.
- Agitation: HY-4 orbital vibrator.
- Weighing: Ohaus Model PA213 analytical balance.

### 2.3 Biosorbent preparation

Coconut husk was collected from mature trees (25–30 years) in Ogugu Town, Awgu LGA, Enugu State, Nigeria. The material was sun-dried, ground in an electric mill, and sieved to a uniform 250 µm particle size fraction using an electromagnetic sieve shaker. No chemical modification or thermal activation was applied. The biosorbent was stored in a sealed container until use. The 250 µm fraction was selected to maximize specific surface area and reduce intra-particle diffusion path lengths (Bayuo et al., 2022; Gupta et al., 2021).

### 2.4 Preparation of metal ion solutions

Stock solutions of Pb(II), Cu(II), and Zn(II) at 1,500 mg/L were prepared as follows:

- Pb(II): 2.397 g Pb(NO_3_)_2_ dissolved in 1,000 mL distilled water.
- Cu(II) and Zn(II): 5.895 g CuSO_4_ 5H_2_O and 6.594 g ZnSO_4_ 7H_2_O were each dissolved in 1,000 mL distilled water.

Working solutions at 300, 600, 900, 1,200, and 1,500 mg/L were prepared by serial dilution. All solutions were freshly prepared to minimize speciation changes. Each metal was studied in a separate single-metal system to isolate individual adsorption behaviour without competitive interference.

### 2.5 Biosorbent characterization

#### 2.5.1 Scanning electron microscopy (SEM)

SEM micrographs were obtained at 1,500×, 3,000×, and 5,000× before and after adsorption to assess surface morphology, porosity, and textural changes associated with metal uptake (El-Naggar et al., 2018; Rao, 2021).

#### 2.5.2 Fourier-transform infrared spectroscopy (FTIR)

FTIR spectra were collected over 400–4,000 cm^-1^ (focus on 650–3,850 cm^-1^) before and after adsorption using KBr optical windows. FTIR identifies characteristic functional groups (–OH, –COOH, C=O, C–O–C) and reveals shifts in peak position or intensity indicative of metal binding (Malik et al., 2017; Rao, 2021).

### 2.6 Batch adsorption experiments

All batch experiments were performed at room temperature (300 K) in 100 mL transparent plastic bottles with a working volume of 20 mL. After equilibration, suspensions were filtered through Whatman No. 1 filter paper, and residual metal concentrations were determined by AAS. Each experimental point was performed in triplicate, and results are reported as mean ± standard deviation (SD). The relative standard deviation was <5% across all replicates.

Unless otherwise stated, variables were investigated individually:

#### 2.6.1 Effect of solution pH

The pH effect was studied over 2.5–6.5 (target values: 2.5, 3.5, 4.5, 5.5, 6.5), adjusted with 0.1 M KOH or HCl. Initial metal concentration was 1,500 mg/L, biosorbent dose 1.0 g per 20 mL, and contact time 120 min. The upper limit was set at 6.5 to avoid metal hydroxide precipitation; thermodynamic calculations (Visual MINTEQ) confirmed that Pb(OH)_2_, Cu(OH)_2_, and Zn(OH)_2_ do not precipitate below pH 6.8 at 1,500 mg/L, ensuring that removal reflected true surface adsorption.

#### 2.6.2 Effect of initial metal ion concentration

Concentration effects were investigated at 300, 600, 900, 1,200, and 1,500 mg/L. The pH was maintained at 4.3 (the natural pH of the stock solution, unadjusted) to isolate the concentration variable. Biosorbent dose was 1.0 g per 20 mL, and contact time was 120 min.

#### 2.6.3 Effect of biosorbent dose

Dose was varied from 0.2 to 1.0 g (0.2, 0.4, 0.6, 0.8, 1.0 g) at a constant metal concentration of 1,500 mg/L, volume of 20 mL, and contact time of 120 min. The pH was maintained at the optimum of 6.0 (identified from the pH study) to maximize removal and ensure comparability across dose levels.

#### 2.6.4 Effect of contact time

Contact time was studied at 5, 10, 20, 30, 40, 50, 60, and 120 min using 1.0 g biosorbent per 20 mL of 1,500 mg/L stock solution. The pH was maintained at the optimum of 6.0 to evaluate kinetics under favourable adsorption conditions. A 120 min contact time was used in all other experimental sets to ensure equilibrium.

### 2.7 Calculation of percentage removal and adsorption capacity

Percentage removal (%R) and equilibrium adsorption capacity (qe, mg/g) were calculated as:

%R=C0​C0‐Ce×100

$$\text{qe}=\text{MV}\left(C0‐\text{Ce}\right)$$

where C_0_ is the initial metal concentration (mg/L), Ce is the equilibrium concentration (mg/L), V is the solution volume ( L), and M is the biosorbent mass (g).

### 2.8 Adsorption equilibrium modelling

#### 2.8.1 Langmuir isotherm

The linearized Langmuir equation:

qeCe=qLkL1+qLCe

where qL (mg/g) is the maximum monolayer capacity and kL (L/mg) is the Langmuir constant. The dimensionless separation factor R_L indicates adsorption favourability:

RL=1+kLC01

Interpretation: R_L > 1 (unfavourable), R_L = 1 (linear), R_L = 0 (irreversible), and 0 < R_L < 1 (favourable).

#### 2.8.2 Freundlich isotherm

The linearized Freundlich equation:

log⁡qe=log⁡KF+n1⁡log⁡Ce

where K_F [(mg/g) (L/mg)^(1/n)] is the adsorption capacity constant and n is the heterogeneity factor. Values of n between 1 and 10 indicate favourable adsorption.

### 2.9 Adsorption kinetic modelling

#### 2.9.1 Pseudo-first-order model

$$\log\left(\text{qe}‐\text{qt​}\right)=\log\text{qe}‐2.303k1\text{​}t\text{​}$$

where qt is the adsorption capacity at time t (mg/g) and k1 is the rate constant (min^-1^).

#### 2.9.2 Pseudo-second-order model

qtt=k2qe21+qet

where k2 is the rate constant [g/(mgmin)]. Model fit quality was evaluated using the linear regression coefficient (*R* ^2^), root-mean-square error (RMSE), and chi-square (χ^2^).

## 3 Results and discussion

### 3.1 Characterization of unmodified coconut husk

#### 3.1.1 Scanning electron microscopy (SEM)

Pre-adsorption SEM micrographs revealed an irregular, rough, and highly porous surface with heterogeneous texture, consistent with the lignocellulosic nature of coconut husk. The porous structure indicates considerable surface area with multiple potential active sites (Duwiejuah et al., 2024; Rao, 2021).

Post-adsorption micrographs showed visible changes: the surface appeared smoother and pores were partially occluded, consistent with occupation of surface sites and pore filling by adsorbed metal ions. These morphological changes confirm successful metal deposition on the coconut husk surface (El-Naggar et al., 2018; Rao, 2021). Similar changes have been reported for other lignocellulosic biosorbents, including coconut husk biochar (Duwiejuah et al., 2024).

#### 3.1.2 Fourier-transform infrared spectroscopy (FTIR)

FTIR spectra revealed several characteristic absorption bands (Table 1). A broad, strong band at 3,432 cm^-1^ (O–H stretching) shifted to 3,429 cm^-1^ after adsorption, with reduced intensity, indicating hydroxyl group participation in metal coordination (Malik et al., 2017; Rao, 2021). The peak at 2,931 cm^-1^ (C–H stretching of aliphatic CH_2_/CH_3_) remained unchanged, suggesting these groups were not involved in binding.

**TABLE 1:** Key FTIR band assignments for coconut husk before and after adsorption.

Wavenumber (cm^-1^)	Assignment	Change after adsorption	Interpretation
3,432 → 3,429	O–H stretching	Shift + reduced intensity	Hydroxyl groups involved in metal binding
2,931	C–H stretching	Unchanged	Not involved
1,633	C=C/O–H bending	Altered intensity	Conjugated structures participate
1,438 → 1,443	C–H bending	Slight shift	Minor contribution
1,256	C–O (aryl ether)	Disappeared	Active site occupation
1,043 → 1,042	C–O–C (ether)	Slight shift	Ether linkage participation
583	Fingerprint region	Disappeared	Structural modification
2,365	CO_2_ (atmospheric)	Appeared	Artifact, not sample-related

The band at 1,633 cm^-1^ (aromatic C=C combined with O–H bending) showed altered intensity after adsorption, indicating participation of conjugated structures. The band at 1,438 cm^-1^ (C–H bending) shifted slightly to 1,443 cm^-1^, suggesting minor contribution. A shift from 1,043 cm^-1^ to 1,042 cm^-1^ (C–O asymmetric stretching of ether linkages) confirms the involvement of oxygen-containing groups (Malik et al., 2017).

The disappearance of peaks at 1,256 cm^-1^ (C–O stretching of aryl/vinyl ethers) and 583 cm^-1^ (fingerprint region) after adsorption further supports multi-site binding. A weak band at 2,365 cm^-1^ observed after adsorption is attributed to atmospheric CO_2_ interference during KBr pellet measurement and is not assigned to sample functionality (Rao, 2021). Collectively, these findings indicate that hydroxyl and ether groups participated in metal binding, consistent with a chemisorption mechanism.

### 3.2 Effect of solution pH on adsorption

**FIGURE 1:**
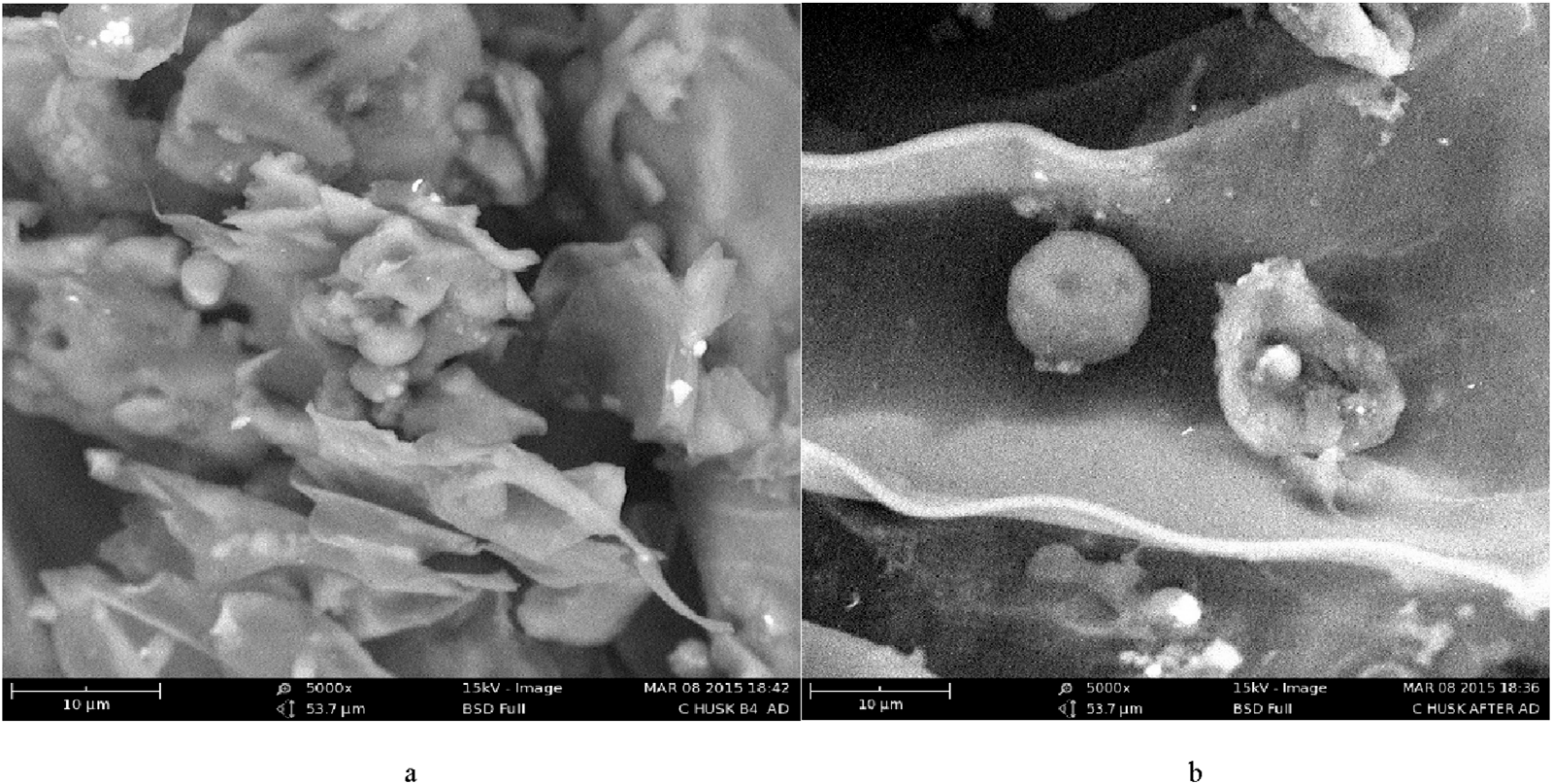
**(a)** SEM image before adsorption of unmodified coconut husk (5000x) **(b)** SEM image after adsorption of unmodified coconut husk (5000x).

The effect of pH was investigated over 2.5–6.5 (Figure 1). For all three metals, percentage removal increased with pH from 2.5 to 6.5, where equilibrium was approached. A sharp increase near pH 5.0 corresponds approximately to the pH of zero charge (pHpzc) of the biosorbent surface, at which the net surface charge becomes negative and favourable for cation attraction (Bayuo et al., 2022; El-Naggar et al., 2018).

At low pH (2.5–4.0), excess protons compete with metal cations for binding sites, suppressing adsorption. As pH increased to 6.5, deprotonation of surface hydroxyl and carboxyl groups increased negatively charged site density, enhancing electrostatic attraction and coordination of metal cations (El-Naggar et al., 2018; Gupta et al., 2021).

Pb(II) consistently showed higher adsorption than Cu(II) and Zn(II) across the pH range. This preferential adsorption may reflect the higher electronegativity and greater affinity of Pb(II) for oxygen-containing ligands, as well as differences in hydrated ionic radii and hydrolysis behaviour (Bayuo et al., 2022; Malik et al., 2017). Based on these results, pH 6.0 was selected as the optimum for subsequent dose and kinetic studies, as it provided high removal efficiency while maintaining a safe margin below the precipitation threshold.

### 3.3 Effect of initial metal ion concentration

**FIGURE 2:**
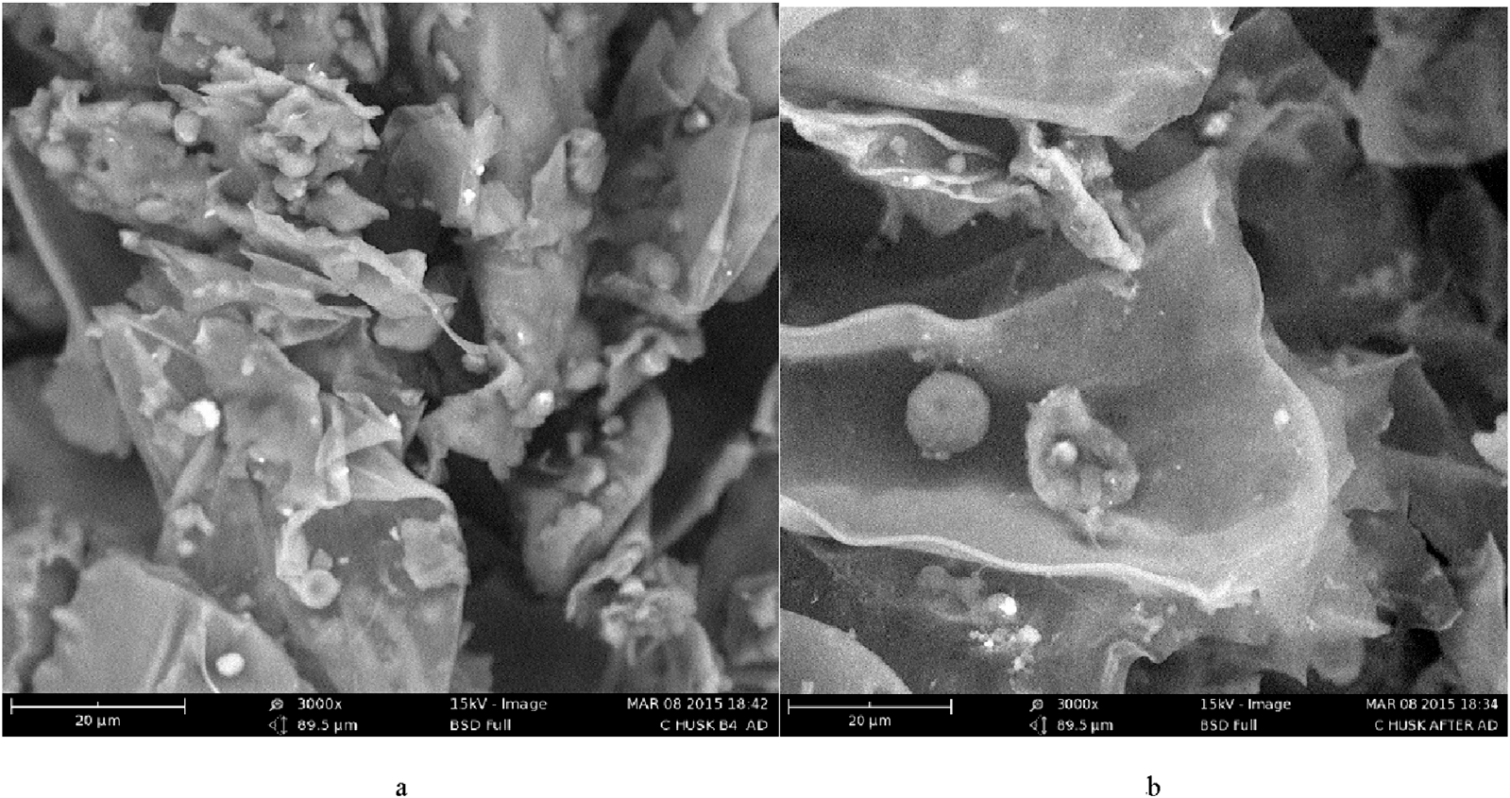
**(a)** SEM image before adsorption of unmodified coconut husk (3000x) **(b)** SEM image after adsorption of unmodified coconut husk (3000x).

The concentration effect was studied at pH 4.3 over 300–1,500 mg/L (Figure 2). Percentage removal decreased with increasing concentration, while adsorption capacity (qe) increased (Table 2). This trend is characteristic of fixed-site biosorbent systems: at lower concentrations, available sites exceed metal ions, yielding high percentage removal; at higher concentrations, site saturation reduces removal efficiency while the concentration gradient increases the mass-transfer driving force (Gupta et al., 2021; Reddad et al., 2002).

**TABLE 2:** Percentage removal and adsorption capacity at varying initial concentrations (pH 4.3, 1.0 g/20 mL, 120 min).

Initial metal ion concentration	Zn(II)	​	Cu(II)	​	Pb(II)	​
​	Percentage removal	Adsorption capacity	Percentage removal	Adsorption capacity	Percentage removal	Adsorption capacity
300	50.63 ± 2.1	3.04 ± 0.13	77.27 ± 1.8	4.64 ± 0.11	95.93 ± 0.9	5.76 ± 0.05
600	41.67 ± 1.9	5.00 ± 0.23	63.64 ± 2.0	7.64 ± 0.24	87.50 ± 1.2	10.50 ± 0.14
900	33.33 ± 2.3	6.00 ± 0.41	55.56 ± 1.7	10.00 ± 0.31	77.78 ± 1.5	14.00 ± 0.27
1,200	25.00 ± 1.8	6.00 ± 0.43	45.45 ± 2.1	10.91 ± 0.50	66.67 ± 1.8	16.00 ± 0.43
1,500	18.75 ± 2.0	5.63 ± 0.60	38.18 ± 1.9	11.45 ± 0.57	53.49 ± 2.1	16.05 ± 0.63

Values are mean ± SD (n = 3).

### 3.4 Effect of biosorbent dose

**FIGURE 3:**
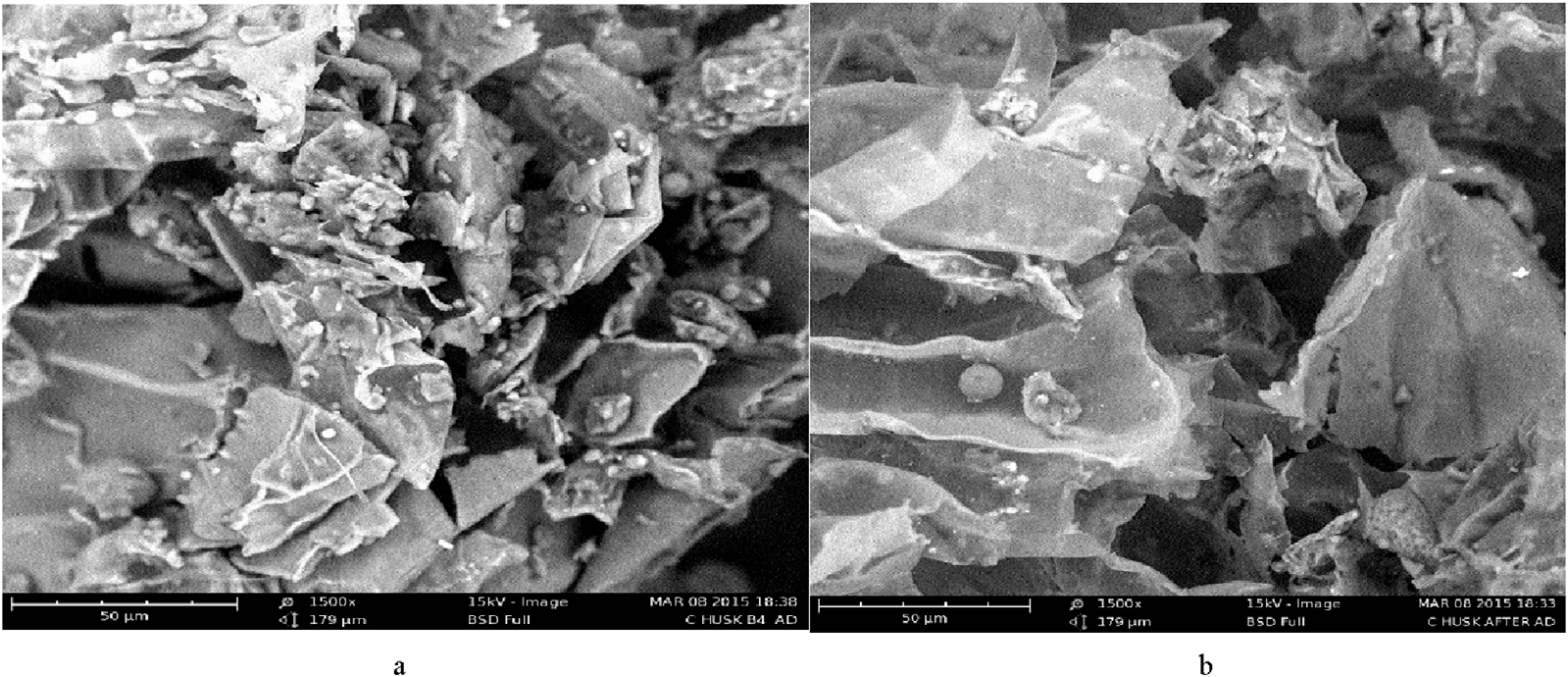
**(a)** SEM image before adsorption of unmodified coconut husk (1500x) **(b)** SEM image after adsorption of unmodified coconut husk (1500x).

Dose was varied from 0.2 to 1.0 g at pH 6.0, 1,500 mg/L, and 120 min (Figure 3). Percentage removal increased with dose for all metals, attributable to the greater total surface area and number of active binding sites available (Duwiejuah et al., 2024; Gupta et al., 2021). Pb(II) showed the widest response range, while Cu(II) and Zn(II) exhibited similar, narrower responses. Although increasing dose improves overall removal, it reduces the adsorption capacity per unit mass because excess sites remain unoccupied (Gupta et al., 2021). A dose of 1.0 g per 20 mL was selected as optimal for subsequent experiments.

### 3.5 Effect of contact time

**FIGURE 4:**
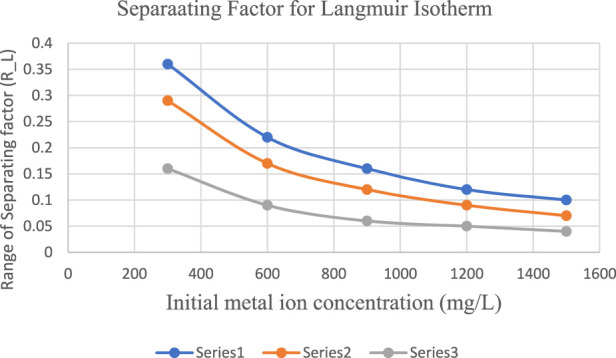
Key: Series 1 = Zn(II), Series 2 = Cu(II), Series 3 = Pb(II).

Contact time was investigated at pH 6.0, 1,500 mg/L, and 1.0 g/20 mL (Figure 4). Adsorption was rapid initially, then progressively slowed as equilibrium approached. Pb(II) attained 69.3% removal within 5 min and reached equilibrium by 40 min. Cu(II) achieved ∼40% removal at 5 min and equilibrium by 40 min. Zn(II) showed an increasing pattern from 5 to 50 min, reaching equilibrium at 50 min with 18.75% removal.

The biphasic profile—rapid initial uptake followed by slower approach to equilibrium—is widely documented for lignocellulosic biosorbents and reflects the sequential occupation of readily accessible external sites followed by slower intra-particle diffusion and saturation of remaining sites (El-Naggar et al., 2018; Gupta et al., 2021). The shorter equilibrium times for Pb(II) and Cu(II) relative to Zn(II) are consistent with their stronger affinity for the oxygen-containing surface groups identified by FTIR. A contact time of 120 min was maintained in all other experiments to ensure full equilibrium.

### 3.6 Adsorption isotherm modelling

#### 3.6.1 Langmuir isotherm

Langmuir parameters are summarized in Table 3. R^2^ values (0.9808–0.9987) indicate excellent fit, supporting monolayer adsorption on a homogeneous surface. The maximum monolayer capacities (qL) followed the order Pb(II) (16.66 mg/g) > Cu(II) (13.05 mg/g) > Zn (6.29 mg/g), consistent with the observed affinity sequence.

**TABLE 3:** Langmuir isotherm parameters (pH 4.3).

Metal	qL (mg/g)	kL (L/mg)	R^2^	RMSE	χ^2^
Zn	6.29	0.0060	0.9985	0.12	0.03
Cu(II)	13.05	0.0079	0.9987	0.18	0.05
Pb(II)	16.66	0.0170	0.9808	0.42	0.11

**TABLE 4:** Langmuir separation factors (R_L) at different initial concentrations.

C_0_ (mg/L)	R_L (Zn)	R_L (Cu(II))	R_L (Pb(II))
300	0.36	0.29	0.16
600	0.22	0.17	0.09
900	0.16	0.12	0.06
1,200	0.12	0.09	0.05
1,500	0.10	0.07	0.04

R_L values (Table 4) for all metals at all concentrations fell within the favourable range (0 < R_L < 1), confirming thermodynamically favourable adsorption.

#### 3.6.2 Freundlich isotherm

Freundlich parameters are presented in Table 5. R^2^ values (0.9520–0.9749) were consistently lower than the corresponding Langmuir R^2^ values for all three metals. This pattern indicates that the Langmuir model provides a superior description of the equilibrium data, supporting the interpretation of predominantly monolayer adsorption rather than multilayer or heterogeneous surface coverage (Bayuo et al., 2022; Gupta et al., 2021).

**TABLE 5:** Freundlich isotherm parameters (pH 4.3).

Metal	K_F [(mg/g) (L/mg)^(1/n)^]	n	R^2^	RMSE	χ^2^
Zn	0.73	3.45	0.9719	0.28	0.09
Cu(II)	0.058	2.87	0.9520	0.51	0.16
Pb(II)	0.051	4.16	0.9749	0.35	0.10

All n values (2.87–4.16) fall within the favourable range (1 < n <10), further confirming favourable adsorption under the tested conditions.

### 3.7 Adsorption kinetic modelling

#### 3.7.1 Pseudo-first-order kinetics

Pseudo-first-order parameters are shown in Table 6. The low R^2^ values (0.5850–0.8762) and the substantial underestimation of qe by the model (1.21–1.40 mg/g vs. experimental values of 5.63–16.05 mg/g) indicate a poor fit. This discrepancy is a well-documented limitation of the pseudo-first-order model for lignocellulosic biosorbents, as it fails to capture the chemisorption-driven rate-limiting step (El-Naggar et al., 2018; Reddad et al., 2002).

**TABLE 6:** Pseudo-first-order kinetic parameters (pH 6.0, 1,500 mg/L).

Metal	qe, calc (mg/g)	k_1_ (min^-1^)	R^2^	RMSE
Zn	1.22	0.016	0.6958	2.41
Cu(II)	1.21	0.021	0.5850	4.89
Pb(II)	1.40	0.022	0.8762	6.83

#### 3.7.2 Pseudo-second-order kinetics

The pseudo-second-order model provided a significantly better fit, with high R^2^ values (0.9694–0.9989) and calculated qe values consistent with experimental data (Table 7). The superior fit implies that the rate-controlling mechanism is chemisorption, involving electron sharing or transfer between metal ions and active surface groups (–OH, C–O–C), which is fully consistent with the FTIR evidence (El-Naggar et al., 2018; Gupta et al., 2021).

**TABLE 7:** Pseudo-second-order kinetic parameters (pH 6.0, 1,500 mg/L).

Metal	qe, calc (mg/g)	k_2_ [g/(mg·min)]	R^2^	RMSE	χ^2^
Zn	5.63	0.0036	0.9694	0.31	0.08
Cu(II)	11.45	0.0233	0.9989	0.14	0.03
Pb(II)	16.05	0.0206	0.9950	0.22	0.05

qe, calc values were reconciled with experimental equilibrium capacities measured at pH 6.0; k_2_ values were derived from linear regression of t/qt vs. t.

## 4 Conclusion

Unmodified coconut husk from Enugu State, Nigeria, is an effective, low-cost biosorbent for Pb(II), Cu(II), and Zn(II) removal from aqueous solution. The material contains abundant oxygen-containing functional groups (hydroxyl and ether) that participate in metal binding via a chemisorption mechanism, as evidenced by FTIR shifts and the excellent fit of pseudo-second-order kinetics. Equilibrium data were best described by the Langmuir isotherm, indicating monolayer adsorption with favourable separation factors. Pb(II) exhibited the highest affinity and capacity, followed by Cu(II) and Zn(II). The rapid initial adsorption kinetics and the use of an unmodified, locally abundant agricultural residue make this biosorbent a promising candidate for low-cost wastewater treatment applications in resource-limited tropical settings.

## Data Availability

The original contributions presented in the study are included in the article/supplementary material, further inquiries can be directed to the corresponding author.
